# Supplementary material for: Direct Binding of the Flexible C-Terminal Segment of Periaxin to β4 Integrin Suggests a Molecular Basis for CMT4F
Source: Front Mol Neurosci. 2019 Apr 9;12:84. doi: 10.3389/fnmol.2019.00084 (PMC6465933; doi:10.3389/fnmol.2019.00084)
Supplement: Supplementary file 1 [file Data_Sheet_1.pdf]

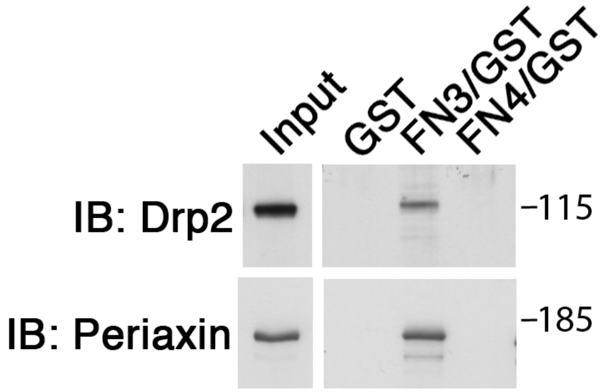

Supplementary Figure S1. β4-FNIII-3 pulls down PRX and the associated DRP2 *in vitro*. GST or a GST-β4-FNIII-3 (FN3/GST) fusion protein were incubated with a sciatic nerve lysate *in vitro* and bound L-PRX or DRP2 were detected by Western blotting (IB). GST-β4-FNIII-4 (FN4/GST) failed to pull down PRX and DRP2.

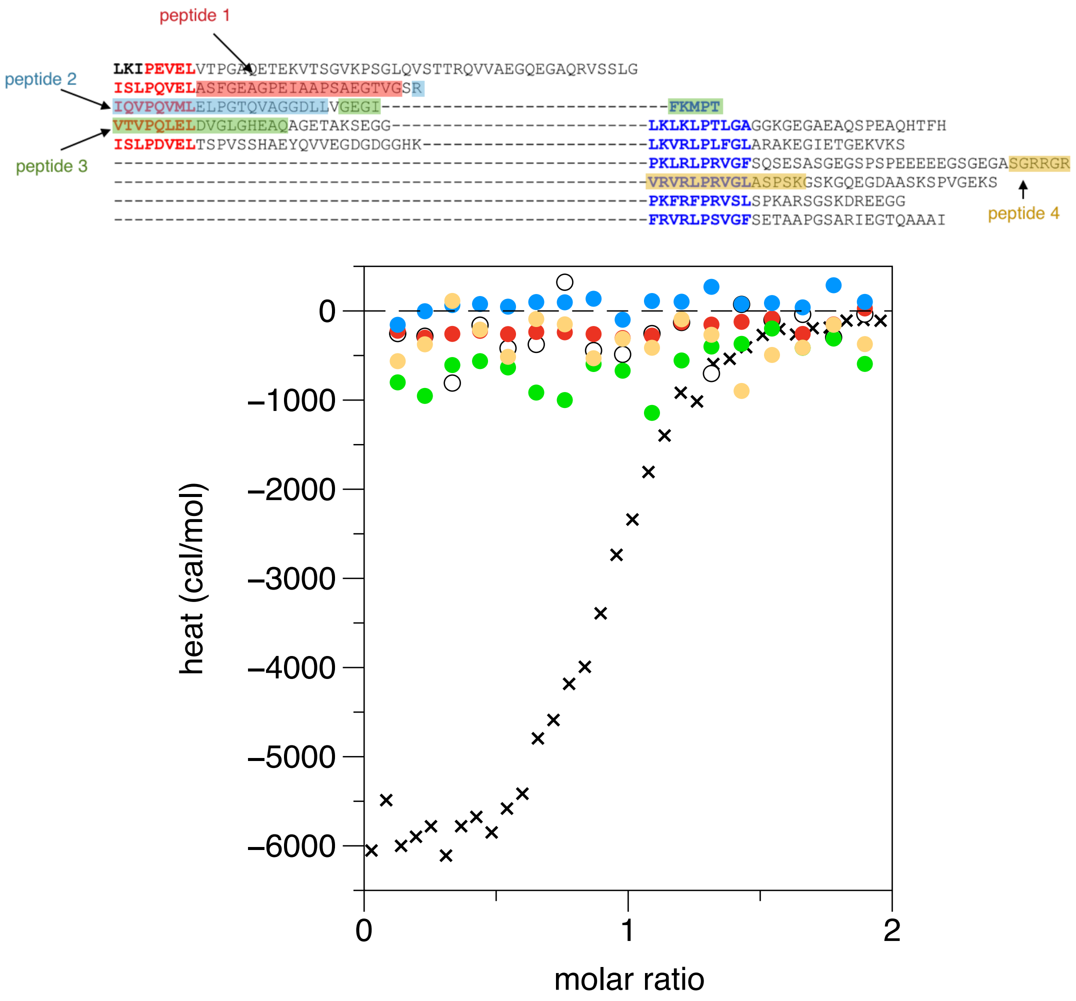

Supplementary Figure S2. ITC screening of potential β4-FNIII-3 binding peptides from L-PRX. Top panel: location of the selected peptides in the PRX-C region (compare to Figure 2B). The peptides correspond to a non-repeat region (peptide 1), a region homologous to a segment of β4-FNIII-3 (peptide 2), an acidic repeat (peptide 3), and a basic repeat (peptide 4). Bottom panel: ITC results. For comparison, the ITC data from the titration with the full PRX-C construct (Figure 2D) is shown (black crosses). Open circles, buffer control. Peptide colours as in top panel. None of the peptides show detectable binding to β4-FNIII-3.

Supplementary Table S1. SAXS modelling. While the MONSA run generated one model using all 3 datasets, the SASREF run performed rigid body fitting of the β4-FNIII-3 crystal structure and the PRX-C GASBOR model to the complex SAXS data.

software	sample	chi^2	data range ($Å^{-1}$)
GASBOR	PRX-C	1.20	0.007-0.1405
MONSA	PRX-C<br>β4-FNIII-3<br>complex	6.49<br>2.51<br>4.58	0.004-0.2454
SASREF	complex	1.46	0.004 - 0.4415
EOM	PRX-C	0.735	0.004 - 0.4415
